# In Situ Gelling Behavior and Biopharmaceutical Characterization of Nano-Silver-Loaded Poloxamer Matrices Designed for Nasal Drug Delivery

**DOI:** 10.3390/gels10060385

**Published:** 2024-06-05

**Authors:** Nadezhda Ivanova, Neli Ermenlieva, Lora Simeonova, Neli Vilhelmova-Ilieva, Kameliya Bratoeva, Georgi Stoyanov, Velichka Andonova

**Affiliations:** 1 Department of Pharmaceutical Technologies, Faculty of Pharmacy, Medical University of Varna, 9000 Varna, Bulgaria; velichka.andonova@mu-varna.bg; 2 Department of Microbiology and Virology, Faculty of Medicine, Medical University of Varna, 9000 Varna, Bulgaria; neli.ermenlieva@mu-varna.bg; 3 Department of Virology, Stephan Angeloff Institute of Microbiology, Bulgarian Academy of Sciences, 26 G. Bonchev Str., 1113 Sofia, Bulgaria; losimeonova@gmail.com (L.S.); nelivili@gmail.com (N.V.-I.); 4 Department of Physiology and Pathophysiology, Faculty of Medicine, Medical University of Varna, 9000 Varna, Bulgaria; k_brat@abv.bg; 5 Clinical Pathology, Complex Oncology Center, 9700 Shumen, Bulgaria; georgi.geesh@gmail.com

**Keywords:** silver nanoparticles, chlorhexidine, Kolliphor P407, thermogelation, phase transition, sol–gel transition, protective nasal spray, respiratory infections, prophylaxis, virucidal activity

## Abstract

A combination of Poloxamer 407 (P407) and hydroxypropyl methylcellulose (HPMC) hydrosols is proposed as an in situ thermo-gelling vehicle for the nasal drug delivery of chlorhexidine–silver nanoparticles conjugates (SN-CX). Optimization of the formulation was carried out by applying varying ratios of P407 and HPMC in the presence and absence of SN-CX so that gelation would occur in the temperature range of the nasal cavity (30–34 °C). Mechanisms for the observed gelation phenomena were suggested based on viscosimetry, texture analysis, and dynamic light scattering. Tests were carried out for sprayability, washout time, in vitro drug release, ex vivo permeation, and antimicrobial activity. When applied separately, HPMC was found to lower the P407 gelation temperature (T_g_), whereas SN-CX increased it. However, in the presence of HPMC, SN-CX interfered with the P407 micellar organization in a principally contrasting way while leading to an even further decrease in T_g_. SN-CX-loaded nasal formulations composed of P407 16% and HPMC 0.1% demonstrated a desired gelation at 31.9 °C, good sprayability (52.95% coverage of the anterior nasal cavity), mucoadhesion for 70 min under simulated nasal clearance, expedient release and permeation, and preserved anti-infective activity against seasonal Influenza virus and beta-coronavirus, *Pseudomonas aeruginosa, Klebsiella pneumoniae, Staphylococcus aureus* and other pathogens. Our findings suggest that the current development could be considered a potential formulation of a protective nasal spray against respiratory infections.

## 1. Introduction

In situ gelling pharmaceutical formulations find broad application as drug delivery systems when prolongation of the retention time at the site of administration is required to improve the therapeutic result. Upon reaching the designated biological compartment, these polymeric systems undergo sol-to-gel transition initiated by different stimuli such as changes in the temperature, pH, ionic environment, or others [1,2,3]. Thereby, they ensure drug immobilization into a highly viscous layer, adherence to the target region, and a sustainable effect. The usage of in situ gelling vehicles is highly appropriate in the development of nasal dosage forms for local, systemic, or nose-to-brain drug delivery [1,4,5,6]. 

The protective nasal formulations are a subject of growing interest in this post-COVID-19 pandemic period [7]. Since society’s self-awareness of complying with the public health culture principles was provoked, scientists have become more and more engaged in developing new, alternative, and more efficient ways for reducing the spread of infectious respiratory diseases [8]. The protective nasal sprays may exhibit different mechanisms of action, but, in general, they concentrate on supporting the natural defense capacity of the nasal mucosa, enhancing the biophysical barrier function of the mucus, and/or exerting microbicidal activity at the site of the pathogen’s entry [9]. An effective protective nasal formulation is required to possess good sprayability; to form stable, even, and retentive film on the mucosa; to not harm the nasal cilia; and to preserve the active ingredients’ activity by ensuring physical and chemical stability, compatibility, and adequate drug liberation [1,9]. 

The thermo-sensitive Poloxamer 407 (poly(ethylene oxide)-poly(propylene oxide)-poly(ethylene oxide) triblock copolymer)—P407—was selected as the main constituent in the composition of in situ gelling nasal spray for protection against respiratory infections. The polymer is known for its low toxicity, good tolerability, great chemical inertness, and drug–polymer compatibility [2,10]; because of its amphiphilic nature, P407 self-assembles into micelles, which aggregate with the increase in temperature and forms a crystal network (micelles order into a hexagonal crystalline packing and form a solid gel) [11,12]. The gelation temperature (T_g_) of P407 solutions depends on the polymer’s concentration and the presence of additives such as active pharmaceutical ingredients and excipients [11]; in any case, the gelation of P407 ≥ 15 *w*/*w*% aqueous solutions occurs in a temperature range appropriate for biomedical applications [13,14]. 

Often, P407 is combined with other polymers in order to improve the formulation’s adhesiveness and gel strength (e.g., cellulose derivatives, chitosan, carbomer, and poly(vinyl)pyrrolidone) [14,15]. In this study, we focused on hydroxypropyl methylcellulose (HPMC)—a polymer with thermoreversible gelation manifesting at much higher than physiological temperature (>65 °C) but with good bioadhesive properties [14,16,17]. It was hypothesized that a P407-HPMC combination could be obtained with eligibility for nasal application, adhesive properties, low viscosity, good sprayability at the ambient temperature, and gelation point in the temperature range of the nasal cavity (30–34 °C) [4,18]. 

Ultimately, this study aimed to result in the establishment of a suitable vehicle for the nasal delivery of chlorhexidine–silver nanoparticles conjugates (SN-CX) as an active ingredient. SN-CX were previously synthesized in our laboratories and characterized with explicit antimicrobial properties against Influenza Type A, *Staphylococcus aureus, Escherichia coli*, and *Candida albicans* [19]. Our ongoing research follows the activity of SN-CX against these and other respiratory pathogens (seasonal beta-coronavirus, *Pseudomonas aeruginosa,* and *Klebsiella pneumoniae*) when in the composition of the co-polymeric solution and the biopharmaceutical characteristics of so-obtained nasal spray. Although several research papers have reported the inclusion of silver nanoparticles (SN) into P407-based thermo-gelling vehicles [20,21,22,23,24,25], the mechanisms by which nano-silver colloids interfere with the P407 micellization and gelation have not yet been widely explored. We attempted a stepwise investigation of the SN-CX impact on the micellar arrangement and point of solidification of P407 hydrosols and combined P407-HPMC hydrosols.

## 2. Results and Discussion

### 2.1. Optimization of the In-Situ Gelling Nasal Composition Based on Viscosimetry, T_g_, Textural, and Colloidal Properties

#### 2.1.1. Viscosimetry

The viscosimetry revealed an increase in viscosity and a sol–gel transition with the rise in temperature up to 45 °C for all test formulations; exceptions were made for the HPMC 0.5% solutions, whose inherent temperature-dependent viscosity was not significantly affected by the presence of SN-CX (within the test temperature range), and the P407 15.5% solutions, combined with HPMC. P407 20% hydrosol was found to undergo gelation at 22.7 °C, while P407 20% hydrosol containing conjugates of silver nanoparticles with chlorhexidine (SN-CX) solidified at 26.1 °C. There are very few reports in the literature that we could relate this result to. Although widely formulated within Poloxamer-containing drug delivery vehicles, to the best of our knowledge, neither form of silver nanoparticles has yet been investigated with respect to interaction with the polymer’s gelation. However, several surveys on other types of nanoparticles in Poloxamer formulations and their effects on gelation are available, and expectedly, they report divergent trends [26]. For example, poly(isobutyl cyanoacrylate) (PIBCA) nanoparticles coated with a mixture of chitosan and thiolated chitosan did not show substantial influence on P407 gelation in the study of Pradines et al. [27]; Laponite-silicate nanoparticles [28], chitosan nanoparticles [29], and ZnO nanoparticles [30] were demonstrated to lower T_g_ and/or improve the strength of poloxamer gels, while solid lipid nanoparticles (SLN) increase the T_g_ [31].

HPMC 0.1%-enriched P407 solutions showed a decrease in T_g_ as compared to pure P407 solutions with the same concentration. These findings are in accordance with other reports from original studies on the subject [2,14,32,33].

**Figure 1:**
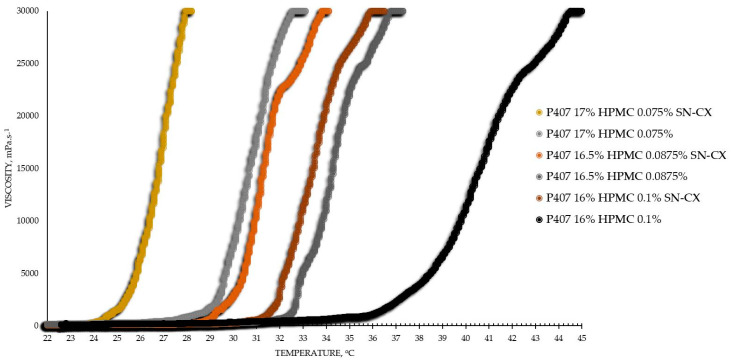
Viscosity–temperature curves of SN-CX-loaded and drug-unloaded P407-HPMC test formulations.

Based on the results from this primary screening, the test formulations were obtained by mixing stock solutions of both hydrosols—P407 20% and HPMC 0.5%—in varying ratios; upon this procedure, formulations with gradiently dropping P407 and raising HPMC concentrations were yielded. As the proportion of the HPMC hydrosol in the formulation increased, expectedly, gelation was observed at higher temperatures due to the stepwise dilution of the P407 hydrosol [33]. Interestingly, when SN-CX was present in the mixtures, a lower gelation point was recorded at all P407-HPMC ratios (Figure 1). 

As a practical contribution of this analysis, the formulation P407 16% HPMC 0.1% SN-CX was selected as an appropriate in situ gelling formulation for nasal administration because of its favorable T_g_ at 31.9 °C; the temperature in the nasal cavity is reported to be in the range of 30–34 °C [4,18]. Furthermore, the formulation in question was characterized as suitable for spaying low viscosity at room temperature (120 mPa.s, Table 1) [4].

**Figure 2:**
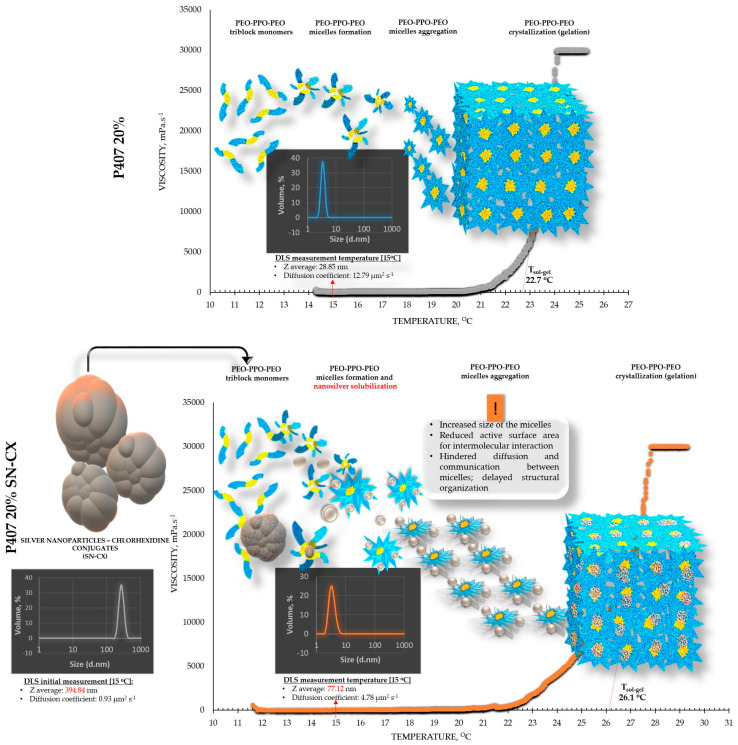
Speculative illustration of the mechanism of gelation of P407 sols and SN-CX-loaded P407 sols.

**Figure 3:**
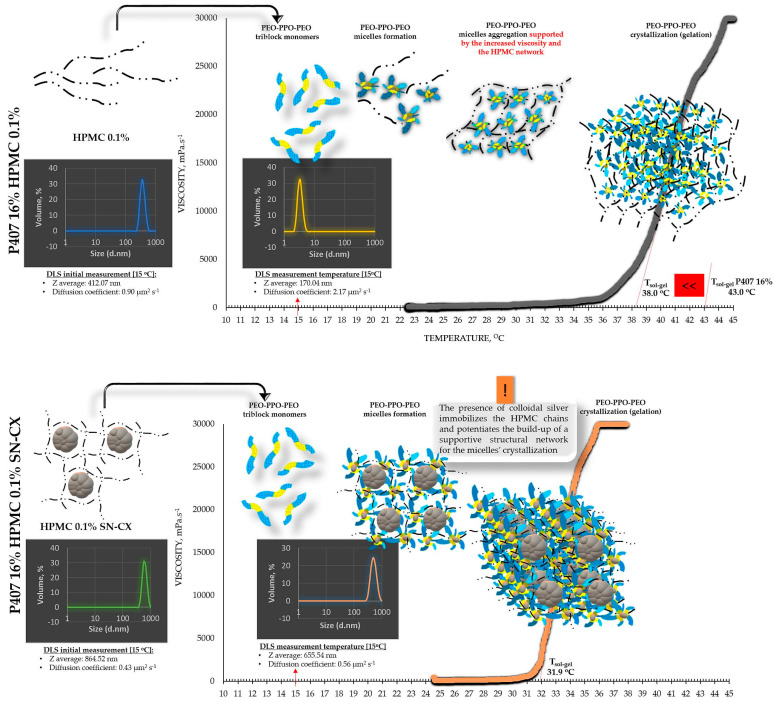
Speculative illustration of the mechanism of gelation of P407-HPMC combined sols and SN-CX-loaded P407-HPMC combined sols.

From an analytical point of view, we found that separately, SN-CX increased the T_g_ of P407, and HPMC decreased it. Still, when applied to a combined P407-HPMC solution, the colloidal particles further potentiated the gelation and decreased the T_g_. To investigate these phenomena, a DLS analysis was performed, and a mechanism for the occurrence of this controversy was proposed (Figure 2 and Figure 3).

#### 2.1.2. Dynamic Light Scattering (DLS)

DLS analysis was carried out for all single components and mixtures of interest. A decision was made to perform the tests at a temperature low enough to ensure a sol state of all samples—15 °C. The results from the front angle (12.78°) scans were considered as they demonstrated eligibility for comparative purposes for all materials. Under these conditions, the hydrodynamic diameter (d_H_, Z-average, nm) of the colloidal SN-CX conjugates was found to be 394.84 ± 42.86 nm; visibly, d_H_ drastically decreased when the nanoparticles were introduced into the P407 20% solution (77.12 ± 5.80 nm), thus suggesting micelle-assisted solubilization and localization amongst the hydrophilic P407 regions [34,35]; solubilization in the hydrophobic micellar cores is unlikely because of the prevailingly hydrophilic nature of the superficial silver ions and chlorhexidine diacetate. On the contrary, when the same concentration of SN-CX was included in an HPMC 0.5% solution, a more than two-fold increase in d_H_ (864.52 ± 60.39 nm) revealed adsorption of the negatively charged polymer (−) onto the positive (+) colloidal surface of SN-CX (ζ = +44.59 [19,36]). Indeed, the visual appearance of both SN-CX-carrying solutions was compliant with this result since P407 20% SN-CX appeared as a fully transparent yellow solution, while HPMC 0.5% SN-CX possessed an orange-brown color and a slight opalescence. When SN-CX was placed in a combined solution of P407 16%-HPMC 0.1%, the obtained d_H_ values of 655.54 ± 71.16 nm eloquently testified that the silver nanoparticles’ solubilization is limited by the HPMC shield on the particles’ surface and their respective enlargement.

The colloids’ diffusion coefficients were derived because of their straight relation to the particles’ mobility, interaction, and ability to assemble into organized structures (determining the point of gelation); they were all found to correspond well with the average hydrodynamic size in the system. Because of the complexity and heterogeneity of the colloidal mixtures, d_90_ and span values were also considered; the first parameter indicates the size limit within the predominant part of 90% of the sample, while the latter is a measure of the size distribution within the sample (span = (d_90_ − d_10_)/d_50_). The interpretation of d_50_ and d_10_ is analogous with d_90._ Although all test formulations were of a complex nature and obtained by mixing different-sized colloids, the resulting samples exhibited a low span value of <0.7, indicating a relatively narrow size distribution within the sample’s volume. All data from the DLS analysis are presented in Table 1.

Based on the viscosimetry and DLS results, the following suggestions were made to explain the observed gelation phenomena: 1. When SN-CX are added to P407 hydrosol, they are shown to solubilize and therefore lead to an increased size of the micelles; thus, the active surface area for intermolecular interaction is reduced, the diffusion and communication between the micelles is hindered, delayed structural organization and gelation at higher temperature is observed; 2. When HPMC is added to P407 hydrosol, the latter crystallizes at a lower temperature, likely due to an increase in viscosity and structural support of the micellar organization by the HPMC chains [14,32]; 3. When SN-CX are added to P407-HPMC combined hydrosols, our results suggest the occurrence of two events: a. the negatively charged HPMC adsorbs on the positively charged SN-CX and prevents their micellar solubilization; b. the presence of colloidal silver immobilizes the HPMC chains and potentiates the build-up of a supportive structural network for the micelles’ crystallization.

#### 2.1.3. Texture Analysis

**Figure 4:**
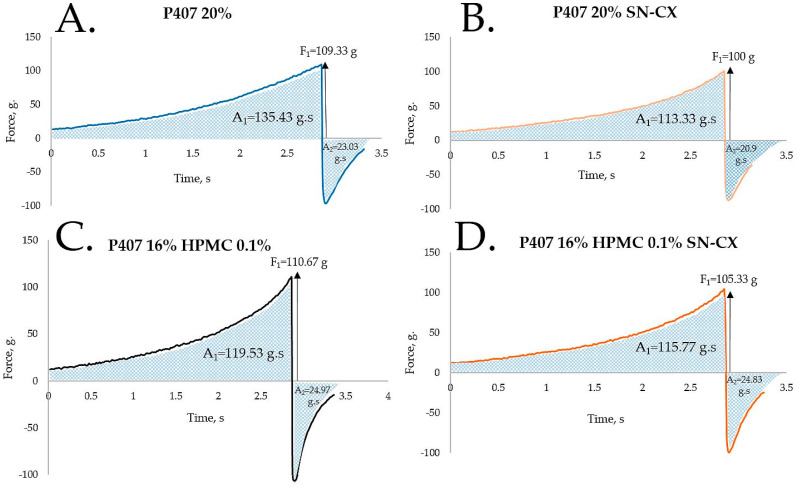
Texture analysis of (**A**) P407 20% hydrogel, (**B**) P407 20% SN-CX hydrogel, (**C**) P407 16% HPMC 0.1% hydrogel, and (**D**) P407 16% HPMC 0.1% SN-CX hydrogel at 34 °C.

HPMC was chosen as a second polymeric ingredient in the thermo-gelling nasal vehicle in order to improve the formulation’s adhesiveness and gel strength. When P407 20% and HPMC 0.5% solutions were mixed in an 80:20 ratio (in order to form a vehicle with an appropriate gelation point—31.9 °C), a final concentration of P407 16% HPMC 0.1% was obtained. Textural analysis of P407 20% and P407 16% HPMC 0.1% in the presence and absence of SN-CX was performed. Although the addition of HPMC solution decreased the P407 concertation from 20% to 16%, it was proven to not only retain but also slightly increase the firmness and adhesiveness of the formed gel at 39 °C while reducing the cohesiveness. The same observation was made in the presence of SN-CX, except for the cohesiveness; the latter was relatively lower for SN-CX-enriched gels as compared to the drug-unloaded gels and was not significantly different for both P407 20% SN-CX and P407 16% HPMC 0.1% SN-CX formulations. This result is likely due to the hypothesized conformational changes in the system caused by the integration of silver nanoparticles in the hydrophilic micellar regions and corresponds with the observed effect of SN-CX on P407 gelation. In addition, the presence of SN-CX weakly reduced the firmness and adhesiveness of both test gels as well, as compared to the drug-unloaded analogs (Figure 4, Table 1).

### 2.2. Sprayability

**Figure 5:**
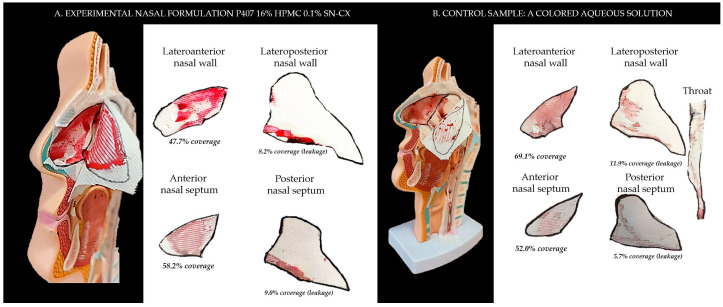
Sprayability of (**A**) P407 16% HPMC 0.1% SN-CX against (**B**) colored water solution, assessed by the area covered (%) in a simulated nasal cavity model: the role of the delivery vehicle.

**Figure 6:**
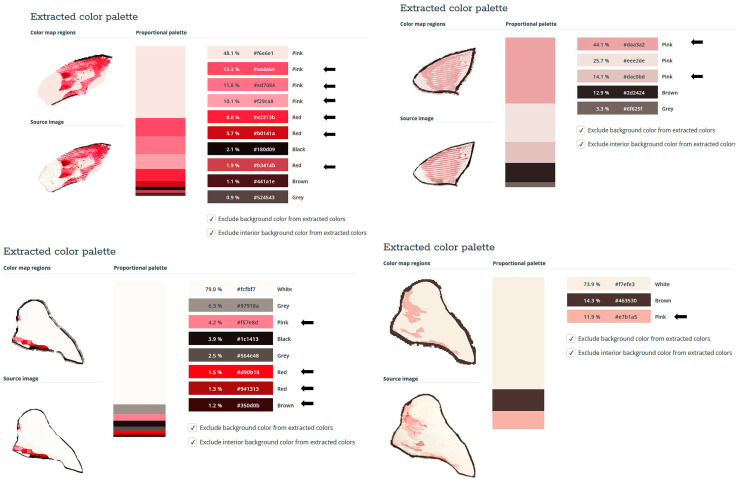
Color analysis of sprayed and dried artificial tissues performed online at https://labs.tineye.com (accessed on 1 February 2024).

The proposed nasal formulation—P407 16% HPMC 0.1% SN-CX—was tested against a water solution in order to follow the influence of the polymeric mixture on the solution’s ability to be sprayed and spread along the anterior nasal cavity. This was carried out using an original methodology for the purpose, and the obtained results testify to its applicability for comparative purposes. Both the test and the control samples were colored in advance with a water-soluble red dye so that the sprayed-on material could be color-analyzed. The loss of sprayability due to an increased viscosity of the test formulation was calculated to be only 12.55% as compared to the performance of pure water injected with the same spraying device. This result was achieved after three-in-a-row injections of the formulations in the nasal cavity model. We should acknowledge that the difference in the covered area between the test and control spray was greater and not as satisfactory when one or two-in-a-row injections were applied. An advantage of the polymeric solution was the better retention on site and the lack of leakage to the throat. It should be noted that the proposed methodology did not allow simulation and consideration of the airflow forces arising upon the recommended sharp inhale while spraying in the nose. We reckon that this method of sprayability testing could be applied as an easy and affordable to reproduce alternative technique in the process of pharmaceutical development; this should be only carried out when better simulation models (such as 3D printed nasal casts or software-assisted models for digital simulations [37,38]) are not available. The results from the sprayability test are presented in Figure 5. Color analysis was performed, as shown in Figure 6.

### 2.3. Washout Time/Mucosal Retention

**Figure 7:**
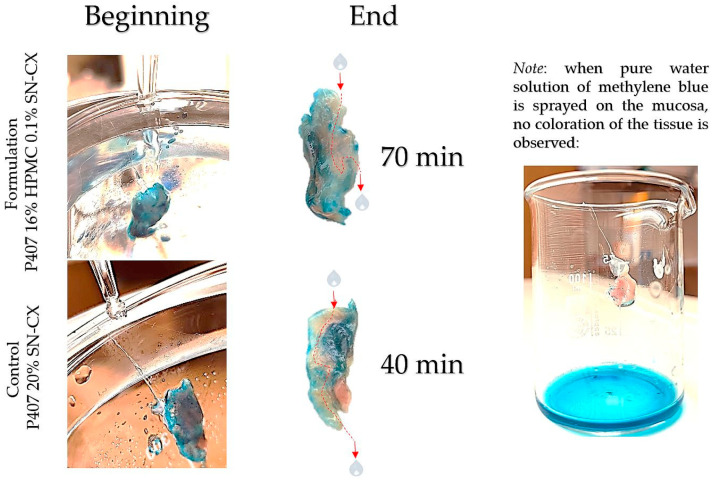
Washout zone and retention time of test formulation P407 16% HPMC 0.1% SN-CX against control formulation P407 20% SN-CX: the role of the mucoadhesive HPMC polymer.

In this study, we aimed to emphasize the role of HPMC in the composition and compared the mucosal retention of the optimized nasal formulation—P407 16% HPMC 0.1% SN-CX—with the one of P407 20% SN-CX. This was carried out upon vertical positioning and fixation of the mucosal tissue and under simulated nasal flow. The time to complete washout was recognized with the aid of methylene blue dye added to the samples in advance. For P407, 20% SN-CX, it was registered to be approximately 40 min, and for P407, 16% HPMC 0.1% SN-CX—70 min. The photographs in Figure 7 demonstrate the beginning and end points of the experiment and the washout zone determined by the path of the drops.

Since warm water (34 °C) was used as a washing fluid in this test and a flow rate comparable to the average physiological nasal secretion debit was maintained (2000 mL per day) [39], we may reckon that the obtained results relate to an extreme case scenario in which the nasal mucus would acquire a viscosity comparable to water under high shear rates typical for sniffing, sneezing, etc. [40].

### 2.4. In Vitro Drug Release and Ex Vivo Mucosal Permeation

**Figure 8:**
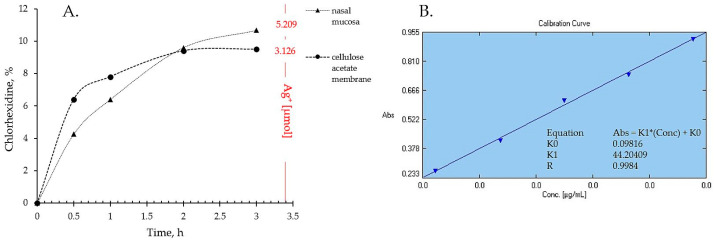
(**A**) Drug liberation and mucosal permeation from P407 16% HPMC 0.1% SN-CX formulation; (**B**) calibration curve of chlorhexidine diacetate in purified water.

The drug liberation from P407 16% HPMC 0.1% SN-CX and the actual permeation through nasal mucosa did not show any notable differences of practical significance. Indeed, the ex vivo study allowed a higher concentration of silver ions in the receptor compartment as compared to the quantity released through the artificial dialysis membrane. Most importantly, neither of the membranes allowed permeation of silver nanoparticles; likely due to the complex structure and the larger hydrodynamic diameter of the nanoparticles in the composition, SN-CX were still undetectable in the receptor media by UV-Vis spectrophotometry after the 3rd hour of the experiments. Despite the broad-spectrum activity, the lack of biodegradation of silver nanoparticles is considered the main disadvantage of this type of nanotechnology [41]. Hence, this result fits a desired case scenario—formulated SN-CX release their active components, i.e., chlorhexidine and silver ions, by dissociation and degradation, respectively, but do not penetrate the mucosa and do not set a prerequisite for cumulation and absorption into the bloodstream (Figure 8).

### 2.5. Antimicrobial Activity

#### 2.5.1. Antibacterial and Antifungal Activity

**Figure 9:**
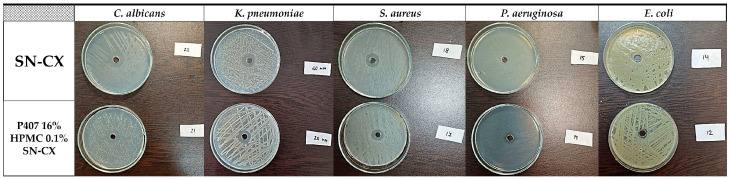
Inhibitory zones of P407 16% HPMC 0.1% SN-CX and SN-CX in seeds of *P. aeruginosa*, *K. pneumoniae*, *S. aureus*, *C. albicans*, and *E. coli*.

The SN-CX conjugates were previously proven with antimicrobial activity against *S. aureus, C. albicans*, and *E. coli*, and minimal bactericidal/fungicidal concentrations were found at 28.6 µg·mL^−1^, 28.6 µg·mL^−1^, and 14.3 µg·mL^−1^, respectively (these values relate to the total active concentration of silver nanoparticles plus the equivalent concentration of chlorhexidine linked) [19]. These results were taken into consideration when choosing the active concentration in the nasal formulation (57.19 µg·mL^−1^). Herein, the antimicrobial potency of SN-CX was assessed within the polymeric solution and compared with the activity of the non-formulated SN-CX colloid. To the test pathogens were added *Pseudomonas aeruginosa* and *Klebsiella pneumoniae* because of their high relevance to respiratory infections and complications [42,43]. Judging by the zones of inhibition on Mueller–Hinton agar, P407 16% HPMC 0.1% SN-CX demonstrated the strongest antimicrobial activity against *C. albicans* (d = 21 mm), followed by *K. pneumoniae* (d = 20 mm), *S. aureus* (d = 17 mm), *P. aeruginosa* (d = 14 mm), and *E. coli* (d = 12 mm). The zones of inhibition obtained with the pure SN-CX solution at the same concentration were found to be either the same or not more than 2 mm wider as compared to the ones achieved with the SN-CX-loaded nasal formulation (Figure 9, Table 2). Such a slightly decreased effect is expected upon the application of viscous dosage forms due to a retarded drug release and diffusion [44].

#### 2.5.2. Antiviral and Virucidal Activity

In order to be able to claim that the effect of the test sample on the cell monolayer during the antiviral experiments is due to viral replication, but not to the cytotoxicity, its cytotoxicity against the cell lines was previously determined. When examining the toxicity of P407 16% HPMC 0.1% SN-CX against the HCT-8 cell line over a period of 120 h (the time required to determine the antiviral effect in the HCov-OC-43 strain), it was found that the test sample was significantly more cytotoxic compared to the reference substance Remdesivir (REM) used (Table 3).

Regarding cytotoxicity evaluation in MDCK cells, P407 16% HPMC 0.1% SN-CX showed considerably higher toxicity than the reference oseltamivir (OS) 450 µg·mL^−1^. These data are in accordance with our previous report on SN-CX toxicity, although, herein, in the polymeric milieu, the toxicity against MDCK cells slightly decreases (Table 4) [19].

When determining the effect on the replication of HCov-OC-43 and IAV A/Panama/07/99 (H3N2), it was found that P407 16% HPMC 0.1% SN-CX, like pure SN-CX, had no effect on this stage of viral reproduction compared to REM (IC_50_ = 12.5 ± 0.9 µg·mL^−1^; selectivity index (SI) = 200.0) and OS (IC_50_ = 1.8 µg·mL^−1^; SI = 250).

The virucidal effect (effect on the viability of extracellular virion particles of corona and influenza virus) of P407 16% HPMC 0.1% SN-CX at its MTC was also investigated. It was found that in the first two incubation time intervals (15 and 30 min), the effect was low and comparable for both viruses (up to 1 Δlg). It slightly increased with the extension of the exposure time, and at 60 min, the viability of HCov-OC-43 and influenza virions was reduced by Δlg = 1.5 and Δlg = 1.25, respectively (Table 5 and Table 6).

The registered virucidal effect against corona and influenza virus is present but weak (compared to standard ethanol 70%) due to the very low MTC of the formulation on HCT-8 and MDCK cell lines. The latter drastically limits the active concentrations to be applied and tested. Still, the obtained results show an increase in the virucidal potency of the nasal formulation in comparison to non-formulated SN-CX [19].

## 3. Conclusions

An in situ gelling vehicle composed of P407 and HPMC for the nasal drug delivery of silver nanoparticles–chlorhexidine conjugates (SN-CX) was developed, and the effects of this active complex on the formulation’s thermo-gelling and mechanical properties were investigated. A DLS-based analysis allowed us to propose a mechanism for the arising phenomena, which, namely, were 1. SN-CX’s undergo solubilization in the P407 sols and suppress the micelles’ crystallization, which leads to an increase in the gelation temperature; 2. HPMC potentiates the P407 gelation, and their mixture acquires lower gelation temperature compared to only P407-containing sols with corresponding concentrations; 3. When SN-CX and HPMC are both present in the composition of the P407 hydrosols, an additional potentiation of the gelation of P407 is observed, and thus even lower gelation temperatures are registered. This interesting finding could be explained by the SN-CX’s significantly enlarged hydrodynamic size when HPMC adsorbs on their surface and the subsequent lack of micellar solubilization. Because of these findings, we hypothesized that instead of acting as a conformational obstacle for the micelles’ organization, SN-CX structurally supports the HPMC-mediated intermolecular communication within the P407-HPMC solutions.

Within the scope of this study, we propose two original methodologies for comparative sprayability assessment and comparative mucosal retention time assessment. Both methodologies are easily reproducible and do not require specific and expensive equipment. Therefore, we consider they could be used as accessible alternatives in the stages of preliminary nasal dosage forms development.

The optimized drug-loaded P407 16% HPMC 0.1% SN-CX test nasal formulation possessed several desirable qualities for its designation: a gelation temperature at 31.9 °C; a better adhesiveness and mucosal retention as compared to P407 compositions without HPMC; a good sprayability ensuring a 52.95% coverage of the anterior nasal cavity; an effective Ag^+^ and chlorhexidine release and mucosal permeation to which we devote the established antimicrobial activity against *Pseudomonas aeruginosa, Klebsiella pneumoniae, Staphylococcus aureus*, *Escherichia coli*, and *Candida albicans* and the weak but present after the 45th minute of contact virucidal effect on influenza and coronavirus virions. According to this study, the proposed formulation has shown eligibility for in situ gelling protective nasal spray on all aspects. As a prospect, an improvement might be sought in terms of lower colloidal size and an even less pronounced shielding effect on antiviral activity.

## 4. Materials and Methods

### 4.1. Materials

Silver nitrate (>99.9%), sodium hydroxide (>98%), and ammonium thiocyanate (>98%) were supplied from Thermo Fisher Scientific, Oxford, UK. Chlorhexidine diacetate salt hydrate (≥98%, Mw 625.55 g/mol) was purchased from Sigma Aldrich, Burlington, MA, USA; Kolliphor^®^ P407, hydroxypropyl methylcellulose (HPMC) (80–120 cps), and Ammonium iron(III) sulfate dodecahydrate were obtained from Sigma-Aldrich, St. Louis, MO, USA; all organic solvents were supplied from Sigma-Aldrich, St. Louis, MI, USA in analytical grade.

### 4.2. Silver Nanoparticles–Chlorhexidine Conjugates (SN-CX)

Aqueous solutions of AgNO_3_ at 2 mM were reduced with green tea-derived phenolic fraction, and silver nanoparticles were obtained; further, the latter were conjugated with chlorhexidine diacetate (CX) following an already established procedure [19,43]. The obtained colloidal solutions were purified by a dialysis method and standardized with a concentration of 700 µg·mL^−1^ nano-silver and 1130 µg·mL^−1^ conjugated CX [19]. The total active concentration of the stock SN-CX solution was considered the sum of SN and CX—1830 µg·mL^−1^. SN-CX samples were stored at room temperature under light protection and used within a safe period of 7 days [45].

### 4.3. Preparation of In Situ Gelling Test Formulations

A stock solution of P407 20% *w/w* was prepared by dispersing the polymer in purified water under static conditions, keeping the so-obtained mixture in a refrigerator for at least 24 h, and gentle homogenization thereafter. Where needed, P407 16% *w*/*w* solution was prepared from P407 20% *w*/*w* stock solution by proper dilution.

A stock solution of HPMC 0.5% *w*/*w* was prepared by dispersing the polymer in preheated purified water at 80 °C under continuous stirring, keeping the so-obtained mixture in a refrigerator for at least 24 h, and homogenization thereafter.

Stock solutions of SN-CX-enriched hydrosols with a concentration of 57.19 µg·mL^−1^ were prepared by the addition of SN-CX stock solution (3.13% *w*/*w*) to previously obtained with a re-calculated quantity of purified water hydrosols of P407 or HPMC so that the final polymeric concentration would be the same as for the drug-unloaded hydrosols—20% *w*/*w* for P407 and 0.5% *w*/*w* for HPMC, respectively.

All polymeric stock solutions were stored in a refrigerator before their use. The drug-unloaded test formulations were obtained by mixing stock solutions of P407 20% *w*/*w* and HPMC 0.5% *w*/*w* in varying ratios—90:10, 85:15, 80:20, and 75:25. SN-CX-loaded test formulations were obtained likewise by using SN-CX-enriched P407 20% *w*/*w* and HPMC 0.5% *w*/*w* stock hydrosols.

All samples that were subjected to antimicrobial studies were prepared under aseptic conditions in a laminar-flow box (UVT-B-AR, DNA/RNA UV-cleaner box, BIOSAN, Riga, Latvia) and with the aid of previously sterilized (autoclaved; 121 °C, 30 min) distilled water. All recommendations for aseptic pharmaceutical preparation were met.

### 4.4. pH Measurement

The pH of the test formulations was measured using Filtres Fioroni pH indicator paper (Filtres Fioroni, Yorkshire, UK), which has a pH measurement range of 5.0–9.0.

### 4.5. Viscosimetry

**Figure 10:**
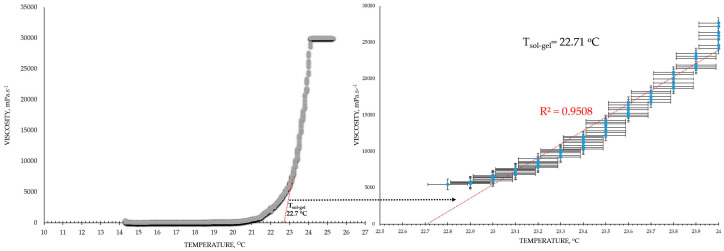
Mathematical extrapolation of the gelation temperature (T_g_) based on the gelation curve’s tangential [46,47,48].

Viscosity analysis of the test formulations was performed on IKA^®^ Rotavisc lo-vi, IKA^®^-Werke GmbH & Co. KG, Staufen, Germany. Spindle№ 4 (SP-4) was used at a rotation speed of 20 rpm, determining a viscosity range detection up to 30,000 mPa·s. The test samples were introduced in a large volume of 500 mL. The temperature was controlled with the aid of an external water bath, and the change in viscosity as a function of the actual sample’s temperature was plotted. Values were registered up to 45 °C. The obtained data were processed using Microsoft Excel^®^ (Microsoft office 2016 professional plus), and the gelation temperature was determined by the trend line passing the ascending region of the curve at minimum R^2^ = 0.95 (the tangential), as shown in the example in Figure 10.

Exception from this methodology made the viscosimetry of HPMC 0.5% solutions because of their significantly lower viscosity and lack of sol–gel transition in the investigated temperature interval. They were assessed with the aid of spindle SP-1 at 50 rpm, which allowed the best obtainable sensitivity in the expected low viscosity range.

The viscosity at 25 °C was calculated for all test formulations as an average of all viscosity values received in the temperature interval between 24.5 and 25.5 °C during the measurements.

### 4.6. Texture Analysis

The samples of interest were previously filled in appropriate for the test containers and tempered at 34 °C in a climatic chamber, Climatest CH 150, ArgoLab, Arezzo, Italy, for at least an hour. The texture analysis was performed on a Belle texture analyzer (Agrosta Overseas, Serqueux, France). The apparatus was equipped with a conical probe with a diameter of 40 mm. The motion of the probe was set at 3 mm·s^−1^ at pre-test and test runs; the insertion depth was 5 cm [49]. The means of three replicates were considered. The graphs were built based on the average values.

### 4.7. Dynamic Light Scattering (DLS)

Zetasizer Ultra Red, λ = 632.8 nm (Malvern Panalytical Ltd., Malvern, UK) was used for the DSL measurements. Multi-angle dynamic light scattering (MADLS) technique was applied at 15 °C. Data were processed and obtained using ZS XPLORER 3.2.0.84 software. Parameters such as average hydrodynamic diameter (d_H_, Z-average), diffusion coefficient, d_90_, and span were obtained. All measurements were repeated in triplicate.

### 4.8. Sprayability

**Figure 11:**
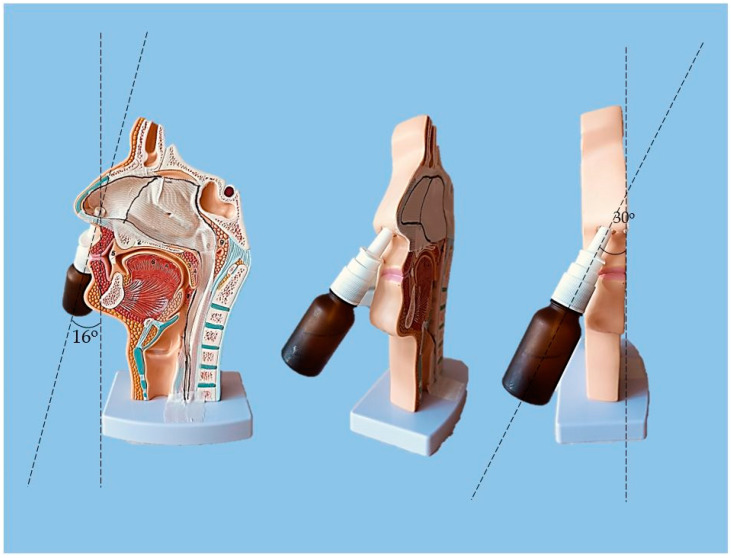
Experimental setting for sprayability testing.

The experimental setting for sprayability testing was built with the aid of a real-sized plastic 3D nasal cavity anatomical model. The surface from the inside was covered with a white synthetic silk surgical tape to allow retention and visibility of the sprayed formulation and removal of the tissue thereafter; the same material was applied as an improvised nasal septum. The four main zones of the nasal cavity, i.e., lateroanterior nasal wall, lateroposterior nasal wall, anterior septum, and posterior septum, were outlined with a black permanent marker. The spraying device was inserted in the nostril and the angles of spraying were determined thereof (Figure 11). The sprayability of the test nasal formulation was assessed against pure water solution; both the test and the control solutions were colored with a water-soluble red dye in advance so that a color analysis could be performed for estimation of the covered area. Each solution was sprayed thrice by using the same spraying device. At least an hour before spraying and right after spraying, the prepared nasal cavity model was kept at 34 °C in a climatic chamber, Climatest CH 150, ArgoLab, Italy. The artificial lining was removed after drying up the sprayed material, cut along the contours, and taped on a blank paper sheet in order for contrast photographs to be taken and color analyzed. The pictures were taken with a realme 11 Pro+ 32 MP camera without magnification. The color analysis was carried out with the aid of the online color extraction platform of TinEye https://labs.tineye.com/color/ (accessed on 1 February 2024). The ability of the formulation to be sprayed was evaluated by the percentage covered area. The loss of sprayability of the test formulation due to the increased viscosity was calculated as follows:

(1) $$\frac{\;Coverage\;of\;the\;lateroanterior\;nasal\;wall,\;\%\;\left(Test\right)+\%\;Coverage\;of\;the\;anterior\;nasal\;septum,\;\%\;\left(Test\right)}{Coverage\;of\;the\;lateroanterior\;nasal\;wall,\;\%\;\left(Control\right)+\%\;Coverage\;of\;the\;anterior\;nasal\;septum,\;\%\;\left(Control\right)}\cdot 100$$

### 4.9. Isolation of Nasal Mucosa Explants

The method for nasal mucosa preparation was selected according to Wadell et al.’s methodology [50]. The tissues were obtained from the porcine snout of a pig weighing 80–90 kg and aged 7–8 months in a certified slaughterhouse (Varna, Bulgaria). The snout was opened by pathologists using forceps and a scalpel, and the pieces of nasal mucosa were isolated from the cavity mucosa and placed on ice during transport to the laboratory. Within 60 min of removal, pieces of nasal mucosa were carefully cut with a scalpel of appropriate size for the mucosal retention test and the ex vivo permeation test.

### 4.10. Determination of Mucosal Retention Time/Washout Time

**Figure 12:**
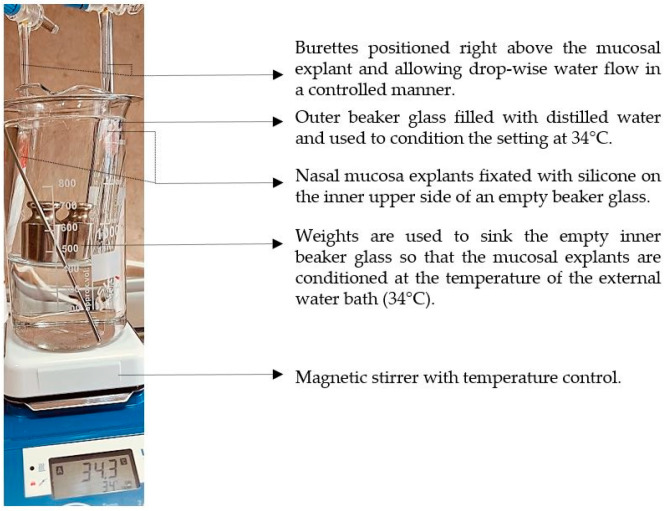
Experimental setting for mucosal retention time testing.

The experimental setting for mucosal retention time was based on vertical fixation of the mucosal explants, spraying of the test formulations, initiation of simulated nasal flow, and detection of the time required for the appearance of a washed-out zone (Figure 12). Subjects to this test were P407-HPMC drug-loaded optimized formulation and HPMC-free equivalent formulation. Both test samples were colored with methylene blue before spraying on the mucosa. A pure water solution of methylene blue was used as a negative control in order to establish the compound’s lack of coloring potential when not in the composition of a viscous and mucoadhesive form. Nasal mucosa explants with an approximate size of 1 × 2 cm were fixated to the upper inner side of an empty beaker glass with the aid of silicone glue. The tissue’s temperature was adjusted to 34 °C by ensuring tight contact with an external water bath, as shown in Figure 12. So prepared, the setting was allowed to condition for an hour before performing the experiment. A single spray of each formulation was applied to the mucosal explants. A simulation of nasal flow was initialized by using warm water (34 °C)-loaded burettes positioned right above the medial axis of the sprayed mucosal pieces. A flow rate of 1.4 mL·min^−1^ (30 drops per minute) resembling the physiological nasal secretion debit (2000 mL per day) was set [39]. The time required for the appearance of a clear washed-out zone was recorded. The mucosal explants were gently dried with the aid of a filter paper at the end of the experiment, and photographs were taken (realme 11 Pro+ 32 MP camera without magnification).

### 4.11. In Vitro Drug Release

The in vitro drug release (permeation) study was carried out on Franz diffusion with a receptor volume of 8.0 mL and an orifice of 0.98 cm^2^ (PermeGear, Hellertown, PA, USA); Spectra/Por^®^ cellulose membrane (MWCO: 12–14 kDa) was used for the purpose. The receptor compartment was filled nearly to the upper limit with distilled water, and the cell was conditioned at 34 ± 0.5 °C with a circulating thermostatic bath. A 0.83 g test hydrosol (corresponding to 1.0 mL formulation at 25 °C) was introduced in the donor compartment, and the receptor volume was adjusted to the top. A stirring rate of 1000 rpm was set. A sample of 0.5 mL was withdrawn on 0.5, 1, 2, and 3 h from the receptor media for quantification of SN-CX and CX. UV-Vis spectrophotometric analysis (at λ = 439 nm for SN-CX and λ = 231 nm for CX [19]) was applied for the purpose, and a standard curve calculation was used. The analyzed sample was returned to the receptor compartment and if an adjustment of the volume to the top was needed, distilled water was used. At the end of the experiment titrimetric assay of silver ions (Ag^+^) was performed by using an adjusted pharmacopoeial method with ferric ammonium sulfate 100 mg·mL^−1^ as indicator and ammonium thiocyanate 0.01 mM as titrant [51].

### 4.12. Ex Vivo Drug Permeation

**Figure 13:**
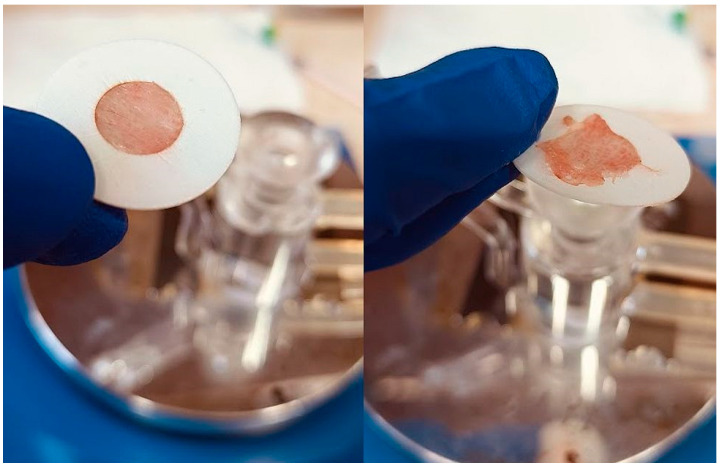
Fixation of the nasal mucosa for the ex vivo permeation study.

The ex vivo permeation study was carried out by using nasal mucosa explant as a membrane on Franz diffusion cell; the applied procedure followed the same specifications as described above (Section 4.11). The mucosal tissue was positioned in the cell with the aid of a fixation ring, as shown in Figure 13. The mucosal integrity was checked before starting the experiment with a bubble test.

### 4.13. Antimicrobial Activity

#### 4.13.1. Antibacterial and Antifungal Activity

A cup–plate technique was applied for evaluation of the antimicrobial activity of SN-CX and SN-CX-loaded test nasal formulation against *Staphylococcus aureus* (ATCC 25923)*, Escherichia coli* (ATCC 25922), *Candida albicans* (ATCC 10231)*, Pseudomonas aeruginosa* (ATCC 10145)*,* and *Klebsiella pneumoniae* (ATCC 10031)—MicroSwabs, Ridacom, Bulgaria. Dense seeds of 0.5 MF standardized microbial suspensions were made on Mueller–Hinton agar (HiMedia^®^, Ridacom, Sofia, Bulgaria). After drying, using a sterile borer, a 7 mm wide well was carved in the center of each Petri’s agar and filled with 100 μL of the test formulation. The Petri dishes were incubated aerobically at 37 °C for 24 h for the bacterial cultures and at 35 °C for 48 h for *C. albicans*; after that, the emerged inhibition zones were measured in diameter.

#### 4.13.2. Cytotoxicity, Antiviral, and Virucidal Activity

Cells

Permanent HCT-8 [HRT-18] (ATCC-CCL-244™, LGC Standards, Middlesex, UK) was kept at 5% CO_2_ and 37 °C in RPMI 1640 (Roswell Park Memorial Institute Medium, ATCC-30-2001) to which L-glutamine 0.3 g·L^−1^ (Sigma-Aldrich, Darmstadt, Germany), Penicillin 100 UI, horse serum 10% (ATCC-30-2021), and Streptomycin 0.1 mg·mL^−1^ (Sigma-Aldrich, Darmstadt, Germany) were added. Madine–Darby canine kidney MDCK (ATCC-CCL-34™) cell line was a gift from Dr. Cyril Bharbesange, National Reference Center for Influenza, Sciensano, Belgium. Cells were grown in DMEM (Gibco, Washington, DC, USA) containing fetal bovine serum (FBS) 5%, (Gibco), sodium bicarbonate 3.7 mg·mL^−1^, HEPES buffer 10 µM (AppliChem GmbH, Darmstadt, Germany), Penicillin 100 U·mL^−1^, and Streptomycin 100 µg·mL^−1^. Both cell lines were seeded at a density of 2.5 × 105/mL at 37 °C in a 5% CO_2_ incubator Thermo Forma 310 (Thermo Fisher Scientific, Waltham, MA, USA), in 96-well plates (Corning^®^ Costar^®^, New York, NY, USA), Amphotericin B 25 µg·mL^−1^ for 24 h until monolayer confluency is reached.

Viruses

Human coronavirus OC-43 (HCoV-OC43) (ATCC: VR-1558) strain was propagated in HCT-8 cells in a RPMI 1640 maintenance solution; the medium was supplemented with horse serum 2%, Penicillin 100 U/mL, and Streptomycin 100 µg·mL^−1^. The cells were lysed by 2 freeze and thaw cycles 5 days after infection; the virus was titrated according to the Reed and Muench formula [52]. The infectious titer of the stock virus was 10^6.0^ cell culture infectious doses 50% in 1 mL (CCID50.mL^−1^).

Allantoic fluid and MDCK-derived seasonal influenza A virus (IAV) strain A/Panama/07/99 (H3N2) (National Center for Infectious and Parasitic Diseases—NCIPD, Sofia, Bulgaria) were used. The infectious titer of the stock virus was T = 10^−5.0^ lg CCID50.mL^−1^ and used as a stock suspension or at a working dose of 100 CCID50 mL^−1^.

Cytotoxicity assay

Cytotoxicity at the 72nd hour on HCT-8 and MDCK cells was evaluated first by visual microscopic observation and then by cell viability assessment after treatment with varying concentrations of test samples following neutral red (NR) dye uptake assay as described previously [53]. As a reference antiviral inhibitor of coronavirus replication, the stock solution of Veklury^®^ (Gilead Science Inc., Cork, Ireland UC) with a concentration of 150 mg·mL^−1^ was used; it was prepared in double distilled water at a final remdesivir (REM) concentration of 8.3 × 10^−3^ M. As a reference antiviral inhibitor of influenza virus replication, oseltamivir phosphate (OS) (Hoffman-LaRoche, Basel, Switzerland) was used.

Antiviral activity assay

The cells were cultivated in 96-well plates. After a confluent monolayer was formed, cell infection with 0.1 mL viral suspension in tenfold falling dilutions was carried out. The non-adsorbed virus was eliminated after an hour, and 0.1 mL/well maintenance medium was added to the cells. The plates were incubated at 33 °C for 5 days for HCoV-OC43 and at 37 °C for 3 days in a 5% CO_2_ atmosphere for IAV. Unintended cells were used as control, cultivated under the same conditions (cells infected with the maximum concentration of the virus demonstrating the maximum cytopathic effect). Microscopic monitoring of the cellular monolayer was used to determine the infectious viral titer. The visually defined cytopathic effect (CPE) was confirmed by NR Uptake Assay [53]. The optical density (OD) of each well was reported at 540 nm in a microplate reader (Biotek Organon, West Chester, PA, USA).

The antiviral activity of the test formulation was evaluated with the aid of a CPE inhibition test. A 100-cell culture infectious dose of 50% (CCID50) in 0.1 mL (containing a different virus strain) was applied to infect a confluent cell monolayer in 96-well plates. The non-adsorbed virus was removed after an hour of virus adsorption for IAV or 2 h for HCov-OC-43, and the test formulation was added in various concentrations; incubation was carried out for 48 h at 37 °C and 5% CO_2_ for IAV, or 120 h at 33 °C and 5% CO_2_ for HCov-OC-43. The CPE was determined using a neutral red uptake assay, and the percentage of CPE inhibition for each concentration of the test sample was calculated according to a protocol described previously [19]. IC_50_ (the 50% inhibitory concentration) was defined as the concentration causing 50% viral replication inhibition as compared to the virus control.

Virucidal Assay

The test formulation was used in its maximum tolerated concentration (MTC) and combined in a 1:1 ratio with 1 mL containing virus (10^5^ CCID50). The samples were stored at room temperature for different time intervals (15, 30, 45, and 60 min). The residual infectious virus content in each sample was determined by the end-point dilution method, and ∆lgs compared to the untreated controls were evaluated.

Statistical analysis

Data were recorded using Gen5^®^ and further processed by Excel^®^ Microsoft. The values of CC_50_ were calculated using non-linear regression analysis (GraphPad Software, San Diego, CA, USA). The values were presented as means ± SD from three independent experiments.

## Figures and Tables

**Table 1:** Viscosity, T_g_, DLS analysis, and textural analysis of the test formulations.

Formulation	Viscosity (25 °C), mPa·s ± SD	T_g,_ °C	DLS at 15 °C			Texture Analysis at 34 °C				pH
			Z-Average, nm ±SD	Diffusion Coefficient, µm^2^·s^−1^ ± SD	d_90_ ±SD	Span ±SD	Firmness, g ± SD	Adhesiveness, g·s ± SD	Cohesiveness, g·s ± SD	
P407 20%	>29 999	22.7	28.85 ± 2.13	12.79 ± 0.83	4.37 ± 0.53	0.47 ± 0.021	109.33 ± 22.09	23.03 ± 7.26	135.43 ± 29.94	6.5
HPMC 0.5%	4.87 ± 0.09	n.a.	412.07 ± 35.26	0.90 ± 0.05	478.28 ± 51.92	0.52 ± 0.022	n.a.	n.a.	n.a.	n.a.
P407 17% HPMC 0.075%	258.31 ± 14.69	29.2	n.a.	n.a.	n.a.	n.a.	n.a.	n.a.	n.a.	n.a.
P407 16.5% HPMC 0.088%	161.45 ± 13.07	32.3	n.a.	n.a.	n.a.	n.a.	n.a.	n.a.	n.a.	n.a.
P407 16% HPMC 0.1%	130.27 ± 2.84	38.0	170.04 ± 20.77	2.17 ± 0.15	4.38 ± 0.53	0.53 ± 0.026	110.67 ± 10.84	24.97 ± 4.58	119.53 ± 14.29	6.0
P407 15.5% HPMC 0.113%	60.00 ± 0.00	>45	n.a.	n.a.	n.a.	n.a.	n.a.	n.a.	n.a.	n.a.
P407 16%	30.53 ± 3.97	43	n.a.	n.a.	n.a.	n.a.	n.a.	n.a.	n.a.	n.a.
SN-CX	n.a.	n.a.	394.84 ± 42.86	0.93 ± 0.06	344.05 ± 42.02	0.50 ± 0.020	n.a.	n.a.	n.a.	n.a.
P407 20% SN-CX	7 011.64 ± 1125.73	26.1	77.12 ± 5.80	4.78 ± 0.27	4.76 ± 0.42	0.67 ± 0.025	100 ± 16.97	20.9 ± 7.50	113.33 ± 17.96	6.0
HPMC 0.5% SN-CX	4.67 ± 0.15	n.a.	864.52 ± 60.39	0.43 ± 0.02	772.28 ± 83.84	0.51 ± 0.020	n.a.	n.a.	n.a.	n.a.
P407 17% HPMC 0.075% SN-CX	1664.15 ± 713.43	25.3	n.a.	n.a.	n.a.	n.a.	n.a.	n.a.	n.a.	n.a.
P407 16.5% HPMC 0.088% SN-CX	147.87 ± 7.83	29.5	n.a.	n.a.	n.a.	n.a.	n.a.	n.a.	n.a.	n.a.
P407 16% HPMC 0.1% SN-CX	120.00 ± 0.00	31.9	655.54 ± 71.16	0.56 ± 0.04	694.93 ± 84.87	0.65 ± 0.029	105.33 ± 5.57	24.83 ± 3.18	115.77 ± 14.14	6.0
P407 15.5% HPMC 0.113% SN-CX	30.00 ± 0.00	>45	n.a.	n.a.	n.a.	n.a.	n.a.	n.a.	n.a.	n.a.

**Table 2:** Inhibitory zones of P407 16% HPMC 0.1% SN-CX and SN-CX in seeds of *P. aeruginosa*, *K. pneumoniae*, *S. aureus*, *C. albicans*, and *E. coli*.

Pathogen	P407 16% HPMC 0.1% SN-CX, mm	SN-CX, mm
*C. albicans*	21	22
*K. pneumoniae*	20	20
*S. aureus*	17	18
*P. aeruginosa*	14	15
*E. coli*	12	14

**Table 3:** Cytotoxicity of the test sample P407 16% HPMC 0.1% SN-CX against the HCT-8 cell line.

Compound	HCT-8 Cell Line	
	CC_50_^a^ Mean ± SD ^b^ [µg·mL^−1^]	MTC ^c^ [µg·mL^−1^]
P407 16% HPMC 0.1% SN-CX	47.5 ^d^ ± 2.8	9.2 ^d^
REM	2500.0 ± 4.3	1000.0

^a^ CC_50_—cytotoxic concentrations 50%; ^b^ SD—standard deviation; ^c^ MTC—maximum tolerable concentration; ^d^ total active concentration silver nanoparticles plus the equivalent concentration of chlorhexidine.

**Table 4:** Cytotoxicity of the test sample P407 16% HPMC 0.1% SN-CX against MDCK cell line.

Compound	MDCK Cell Line	
	CC_50_^a^ Mean ± SD ^b^ [µg·mL^−1^]	MTC ^c^ [µg·mL^−1^]
P407 16% HPMC 0.1% SN-CX	4.68 ^d^ ± 2.8	3.0 ^d^
SN-CX [19]	4.20 ^d^ ± 0.6 [19]	-
OS	450.0 ± 1.3	360.0

^a^ CC_50_—cytotoxic concentrations 50%; ^b^ SD—standard deviation; ^c^ MTC—maximum tolerable concentration; ^d^ total active concentration of silver nanoparticles plus the equivalent concentration of chlorhexidine bound

**Table 5:** Virucidal activity against coronavirus virions strain HCoV-OC-43.

Compound	Δlg			
	15 Min	30 Min	45 Min	60 Min
P407 16% HPMC 0.1% SN-CX	0.25	0.75	1.25	1.50
Ethanol 70%	5.0	5.0	5.0	5.0

**Table 6:** Virucidal activity against influenza virus virions strain A/Panama/07/99 (H3N2).

Compound	Δlg			
	15 Min	30 Min	45 Min	60 Min
P407 16% HPMC 0.1% SN-CX	0.25	0.50	1.00	1.25
SN-CX [19]	0	0	0.33	0.33
Ethanol 70%	4.0	4.0	4.0	4.0

## Data Availability

The data presented in this study are openly available in article.
